# Development and Tracking of Body Mass Index from Preschool Age into Adolescence in Rural South African Children: Ellisras Longitudinal Growth and Health Study

**DOI:** 10.3329/jhpn.v26i4.1882

**Published:** 2008-12

**Authors:** K.D. Monyeki, M.A. Monyeki, S.J. Brits, H.C.G. Kemper, P.J. Makgae

**Affiliations:** 1 Chronic Disease of Lifestyle Unit, Medical Research Council, Tygerberg, 7505, South Africa; 2 School of Biokinetics, Recreation and Sport Science, North West University, Potchefstroom Campus, Potchefstroom, 2520, South Africa; 3 Department of Kinesiology and Physical Education, University of Limpopo, Sovenga, 0727, South Africa; 4 VU University Medical Center, Institute for Research in Extramural Medicine (EMGO), 1081 BT Amsterdam, The Netherlands

**Keywords:** Adolescent, Body mass index, Child, Child growth, Cohort studies, Obesity, Observational studies, Prospective studies, Tracking, South Africa

## Abstract

The purpose of this observational prospective cohort study was to investigate the development and tracking of body mass index (BMI) of Ellisras rural children from preschool age into late adolescence from the Ellisras Longitudinal Growth and Health Study. Heights and weights of children were measured according to the standard procedures recommended by the International Society for the Advancement of Kinanthropometry twice a year from 1996 to 2003. In total, 2,225 children—550 preschool and 1,675 primary school—aged 3-10 years (birth cohorts 1993 to 1986) were enrolled at baseline in 1996 and followed through out the eight-year periodic surveys. In 2003, 1,771 children—489 preschool and 1,282 primary school—were still in the study. The prevalence of overweight was significantly higher among girls (range 1.6-15.5%) compared to boys (range 0.3-4.9%) from age 9.1 years to 14.9 years. The prevalence of thinness (severe, moderate, and mild) ranged from 7.1% to 53.7% for preschool children and from 8.0% to 47.6% for primary school children. Both preschool and primary school children showed a significant association between the first measurements of BMI and the subsequent measurement which ranged from B=0.2 (95% confidence interval [CI] 0.1-0.4) to B=0.8 (95% CI 0.6-0.9) for preschool and B=0.2 (95% CI 0.1-0.3) to B=0.7 (95% CI 0.6-0.8) for primary children. A significant tracking of BMI during 4-12 years of life was more consistent for preschool children (B=0.6 (95% CI 0.6-0.7) and for primary school children (B=0.6 (95%CI 0.5-0.6). Investigation of nutritional intake and physical activity patterns will shed light on how healthy these children are and their lifestyle.

## INTRODUCTION

Increase in childhood obesity is a serious and widespread problem in the world. Statistics from deve-loped and many developing countries suggest that the prevalence of obesity in childhood and adolescence has recently increased drastically, and this trend will continue (1). This is a serious concern as obesity is a prominent risk factor associated with many chronic diseases, such as coronary heart disease, diabetes mellitus, and hypertension (2-5). Despite the growing concerns about childhood obesity, remarkably few analyses on the longitudinal development of obesity in children are available. Longitudinal research is able to depict the association between data recorded at a younger age with the occurrence of diseases at a later stage in life, hence the potential to explore the tracking and stability of a characteristic exists (6).

Tracking or stability of a characteristic is used mostly in relation to the risk factors of chronic diseases (7,8). Early detection of these risk factors can lead to the possibility of early treatment. Quantification of the stability of a characteristic over time is important from a public-health perspective in longitudinal research. Its importance is evident in the effectiveness of lifestyle intervention to improve health. If the stability of a characteristic is very high (close to one), the level of this characteristic is usually hard to change, and therefore, the interventions that focus on these characteristics are predestined to be ineffective (9). Knowledge of the level of tracking of a characteristic further helps answer the question whether or not lifestyle interventions should be provided to the whole population or to a subsample. If the stability is high, the value of a characteristic is a good predictor for the value of this characteristic later in life (8,10). Therefore, in such cases, it is prudent to focus the interventions on those with unhealthy scores because the risk that others will attain unhealthy scores is low. On the one hand, if the stability is extremely low, it will be better to focus on the whole group (11). Furthermore, if tracking exists for a certain risk factor, the subject at risk can be identified at an early age, and, therefore, preventative strategies can start as soon as it is identified at a particular time.

The development of obesity in one of the four critical or sensitive periods—intrauterine life, infancy, the period of adipose rebound (5-7 years), and adolescence—could raise health hazards later in life (6,12-15). An increase in the prevalence of childhood obesity has been reported among black South African children during the current decade since the democratic election in 1994 (16-21). An investigation into the tracking of this endemic remains important in this population. The purpose of this study was, therefore, to investigate the deve-lopment and tracking of body mass index (BMI) of Ellisras rural children from childhood into late adolescence stage from the Ellisras Longitudinal Growth and Health Study (ELS).

## MATERIALS AND METHODS

### Geographical area

Ellisras is a deep rural area situated within the north-western area of the Limpopo province, South Africa. About 50,000 people reside in 42 settlements (22). These villages are approximately 70 km from the Ellisras town (23° 40S 27° 44W), now known as Lephalale, which is adjacent to the Botswana border. The Iscor coal mine and the Matimba electricity power station are the two major sources of employment for many Ellisras residents. The remaining workforce is involved in subsistence farming and cattle rearing while the minority is in the education and civil service. Unemployment, poverty, and low life expectancy seem to play a significant role in the rural South African population, of which the Ellisras rural area people are not an exception (23,24).

### Sample

The ELS initially followed a cluster-sampling method (18). In brief, the study was undertaken at 22 (10 preschools and 12 primary) schools randomly selected from 68 schools within the Ellisras area. Birth records were obtained from the school admission register with the assistance of principals in each school. Only those records that were verified against health clinic records were used for determining the ages of potential participants. Each of the 22 selected schools was assigned a grade with the expectation that most children in a particular age category, i.e. 3, 4,…9, 10, would be found in that grade.

For the purpose of this analysis, data collected in November 1996, May 1997, November 1997, May 1998, November 1998, May 1999, November 1999, May 2000, November 2000, May 2001, May 2002, May 2003, and November 2003 were included. In total, 2,225 children—550 preschool children, mean age 4.4 years, standard deviation (SD)=0.99 and 1,675 primary school children, mean age 8.0 years, SD=1.11—at baseline were followed throughout the periodic surveys. On average, 1.05% of the participants were permanently lost due to death, and 11.47% lost due to teenage pregnancy, illness, and migration to urban areas. School drop-out was a temporary issue as the affected participants rejoined the study thereafter. In total, measurements of 1,771 subjects—489 preschool children, mean age 11.4 years, SD=0.96 and 1,282 primary school children, mean age 14.9 years, SD=1.11—were done in November 2003.

### Anthropometry

All children underwent a series of anthropometric measurements of height and weight according to the standard procedures recommended by the International Society for the Advancement of Kinanthropometry (ISAK) (25). Weight was measured on an electronic scale to the nearest 0.1 kg, and a Martin anthropometer was used for measuring height to the nearest 0.1 cm. From height and weight, the BMI (weight [kg] divided by height [m] squared) was calculated.

### Maturity

The assessment of maturation was included in the anthropometric survey of May 2001 and 2003 for all children who were part of the ELS, and the May 2003 assessment was included in the analysis. Breast development and genital/pubic hair development stage was assessed by visual inspection using Tanner rating scale pictures ranging from 1 (no development) to 5 (matured stage) (26). To reduce embarrassment, older children were provided with a separate private space to complete the self-assessment. Once completed, the self-assessment was verified by visual ins-pection at the ‘skinfold measurements’ station. In instances where the average breast score was between two breast stages, the breast stage was rounded down because the higher breast stage has not been achieved. The palpation of the breast which is the superior method to assess breast development was not possible in the study because it was conducted in a class room as it was included in the ‘skinfold measurements’ station of the anthropometric survey. The qualitative Tanner score was converted into quantitative variables (pubertal stage by Tanner Scale of both sexual organ and breast development): T1=0, T2=0, T3=1, T4=2, and T5=3.

### Quality control

Sixteen anthropometrists carried out the survey over a three-week period each year; these anthropometrists were required to undertake reliability testing as part of their training. This training was conducted by a level three criterion of ISAK following the guidelines of Norton and Olds (25). The absolute and relative values for intra-tester and inter-tester technical error of measurements (% TEM) for weight ranged from 0.12 (0.15%) to 0.31 kg (0.36%) and from 0.22 cm (0.12%) to 0.43 (0.32%) for height.

Well-trained field workers stationed at the ‘skinfold measurements’ station assessed the maturational status. The intra- and inter-tester reliability conducted on 20 subjects (10 boys and 10 girls) who were not part of the survey was 100% in agreement on pubic hair and 92% on breast development.

### Ethics

The Ethics Committee of the University of Limpopo granted ethical approval prior to the survey, and the parents or guardians provided informed consent.

### Statistical analysis

Descriptive statistics of the development over time of absolute body size (height, weight) and BMI were reported. *T*-test was applied to test the significance level between sexes. All the subjects were classified as overweight and obese according to the Cole *et al*. cut-off point (27). The international cut-off points for thinness (grade one, two, and three) by sex for exact ages defined to pass through BMI of 16, 17, and 18 kg/m^2^ were used (28,29). Chi-square test was used for comparing two or more sets of nominal data that have been arranged into categories by frequency counts of large samples while the Fisher's exact test was used when the expected cell frequencies were small (less than five) (30,31). The statistical significance was set at p<0.05.

Partial correlation coefficients controlled for maturation and age were calculated to assess the association between the first measurements of BMI and the follow-up measurements for boys and girls separately. Linear regression model was used for assessing the relationship between BMI at the first measurements and the follow-up measurements adjusted for age and maturation for boys and girls separately.

A longitudinal tracking (generalized estimating equation (GEE) technique which measures the association between an indicator at the first period of measurements and the same indicator at all other periods of measurements was used with maturation and age being included in the model (32-34). Tracking was assessed by calculating odds ratios and 95% confidence interval for subjects “at risk of overweight and thinness (severe, moderate, and mild)” at the initial measurements to maintain ‘at risk of their position’ at the follow-up measurements using the GEE (32-34). All the statistical analyses were done using the SPSS software (version 15) and the Stata program (version 9) (35-36).

## RESULTS

To examine the effects caused by the subjects who were absent, we compared anthropometric measurements with the paired follow-up subjects during each period of measurement. There was significant (p<0.05) difference between subjects who were currently in the study and the drop-out regarding weight, stature, and BMI in the study.

The mean BMIs of primary school girls (mean 15.01, skewness=1.029, kurtosis=2.560) were higher than those of boys (mean 14.629, skewness=0.695, kurtosis=3.050), indicating a more heterogeneous distribution of the sample. The preschool children showed almost a similar mean but different in the sample skewness and kurotsis for girls (mean=13.9, skewness=1.010, kurtosis=4.176) and boys (mean 13.869, skewness=0.359, kurtosis=2.229). The distribution for the preschool girls was normal while it was positively skewed for the primary school girls.

**Fig. 1.**
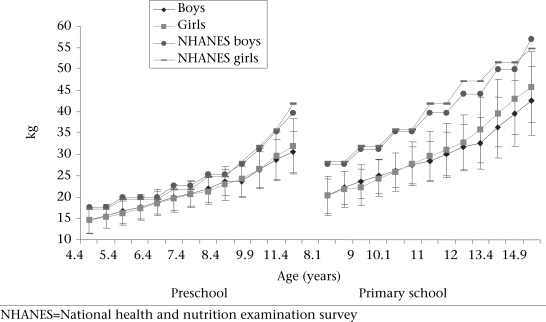
Development of mean weight (kg) by age and sex

**Fig. 2.**
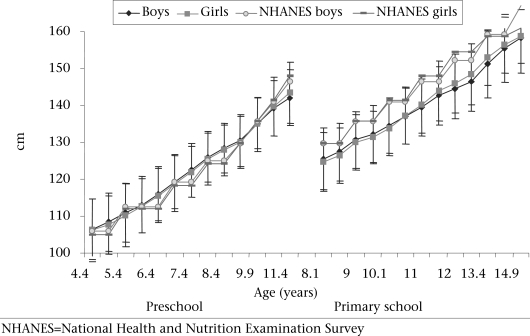
Development of mean stature (cm) by age and sex

**Fig. 3.**
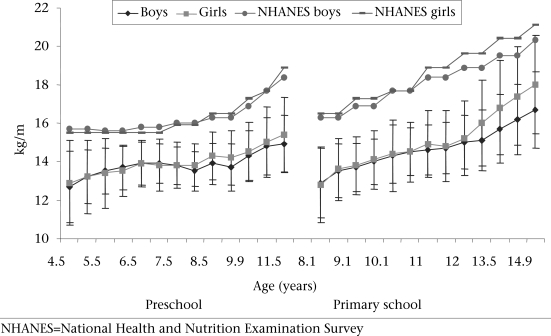
Development of mean body mass index (kg/m^2^) by age and sex

Figure 1-3 present the development of the mean absolute body size and BMI of the Ellisras rural preschool children, primary school children, and the reference population. The BMI and weights of the reference population were higher than those of both preschool and primary school children while the mean stature of the preschool children was close to the mean stature of the reference population for both boys and girls. The primary school girls showed a significant (p<0.05) higher mean stature from 11 to 14.5 years than boys while the preschool and primary school girls showed a significant (p<0.05) higher BMI from 7.9 to 11.5 years and from 11 to 14.9 years respectively compared to boys. The mean BMI for the preschool children was significantly (p<0.001) high in all the overlapping ages of both preschool and primary school children, except between 8.9 and 9.1 and between 9.9 and 9.6 years (Table 1).

**Table 1.** Differences in mean body mass index and frequencies of overweight and thinness for overlapping mean ages for preschool (7.9-11.5 years) and primary school children (8.1-11.6 years) of Ellisras rural children

Overlapping mean age-group (years)		Mean BMI					
		Boys		p value	Girls		p value
Preschool	Primary school	Primary school	Preschool		Primary school	Preschool	
		Mean	Mean		Mean	Mean	
7.9	8.1	12.8	13.8	0.000	12.9	13.5	0.000
8.5	8.5	13.6	14.3	0.000	13.5	13.8	0.002
8.9	9.1	13.8	13.7	0.814	13.7	14.2	0.000
9.9	9.6	14.4	14.3	0.440	14.3	14.5	0.102
10.9	10.6	14.5	14.7	0.012	14.6	15.0	0.000
11.5	11.6	14.7	14.9	0.006	14.8	15.4	0.000
**Preschool**	**Primary school**		**Overweight**				
			**Boys**			**Girls**	
			***x*2 (p value)**			***x*2 (p value)**	
7.9	8.1		2.18 (0.141)			4.42 (0.036)	
8.5	8.5		0.01 (0.932)			0.25 (0.617)	
8.9	9.1		2.49 (0.138*			1.10 (0.294)	
9.9	9.6		3.02 (0.114)*			0.23 (0.631)	
10.9	10.6		0.58 (0.446)			0.34 (0.560)	
11.5	11.6		0.57 (0.468)			1.34 (0.247)	
**Preschool**	**Primary school**	**Thinness**					
		**Boys**			**Girls**		
		**Severe *x*^2^ (p value)**	**Moderate *x*^2^ (p value)**	**Mild *x*^2^ (p value)**	**Severe *x*^2^ (p value)**	**Moderate *x*^2^ (p value)**	**Mild *x*^2^ (p value)**
7.9	8.1	47.88 (0.000)	0.01 (0.93)	18.83 (0.000)	18.95 (0.000)	3.59 (0.06)	5.78 (0.016)
8.5	8.5	28.88 (0.000)	4.48 (0.034)	0.35 (0.554)	10.48 (0.001)	0.18 (0.67)	0.01 (0.913)
8.9	9.1	0.28 (0.597)	1.14 (0.285)	0.03 (0.866)	4.00 (0.045)	2.34 (0.126)	1.20 (0.274)
9.9	9.6	0.62 (0.432)	0.01 (0.945)	0.14 (0.705)	3.63 (0.057)	0.000 (0.995)	0.40 (0.53)
10.9	10.6	0.32 (0.574)	1.05 (0.306)	0.28 (0.596)	0.23 (0.631)	0.50 (0.480)	2.76 (0.097)
11.5	11.6	0.07 (0.797)	3.58 (0.058)	0.01 (0.959)	0.02 (0.900)	0.47 (0.496)	1.44 (0.231)

*x*2=Chi-square test; *Fisher's exact test

Table 2 shows the development of the prevalence of overweight, obesity, and thinness (severe, moderate, and mild) of the preschool (aged 4.5-11.5 years) and primary (aged 8.1-14.9 years) school children. The prevalence of overweight and obesity was low, and the difference between the sexes was not significant. However, the prevalence of overweight among the primary school girls increased to 10.4-15.5% at the age of 13.5-14.9 years, a significant (p<0.05) increase in overweight compared to boys.

**Table 2.** Prevalence of overweight/obesity and undernutrition (for BMI for thinness grade 1, 2, and 3) for preschool (aged 4.5-11.5 years) and primary school (mean age 8.1-14.9 years) of Ellisras rural children

Preschool											Primary school										
	Boys					Girls					Boys					Girls				
Mean age (years)	OVW*	OB*	Thinness†			OVW*	OB*	Thinness†			Mean age (years)	OVW*	OB*	Thinness†			OVW*	OB*	Thinness†		
			SRV	MD	ML			SRV	MD	ML				SRV	MD	ML			SRV	MD	ML
	% (n)	% (n)	% (n)	% (n)	% (n)	% (n)	% (n)	% (n)	% (n)	% (n)		% (n)	% (n)	% (n)	% (n)	% (n)	% (n)	% (n)	% (n)	% (n)	% (n)
4.5	3.7	-	53.7	14.6	14.3	3.9	0.8	48.4	13.4	12.6	8.1	2.2	-	43.6	14.9	23.6	2.7	0.4	37.5	12.2	25.6
	(11)		(158)	(43)	(42)	(10)	(2)	(123)	(34)	(32)		(20)		(393)	(134)	(213)	(21)	(3)	(291)	(95)	(199)
4.9	0.3	-	36.0	18.2	22.6	1.2	1.2	33.9	13.1	25.5	8.5	1.1	-	21.1	18.6	30.9	1.2	0.1	22.1	16.7	31.7
	(1)		(105)	(53)	(66)	(3)	(3)	(85)	(33)	(64)		(9)		(174)	(153)	(254)	(9)	(1)	(165)	(125)	(237)
5.5.	0.7	-	21.8	20.4	33.6	0.8	0.8	25.0	19.6	24.2	9.1	0.3	-	16.1	20.0	37.4	1.6	0.4	16.8	20.0	37.6
	(2)		(61)	(57)	(94)	(2)	(2)	(60)	(47)	(58)		(3)		(139)	(173)	(324)	(13)	(3)	(136)	(162)	(305)
5.9	0.4	-	14.1	19.4	29.7	0.5	-	20.5	16.8	22.7	9.6	0.3	-	10.5	17.4	39.7	1.8	-	14.5	18.0	37.3
	(1)		(37)	(51)	(78)	(1)		(45)	(37)	(50)		(2)		(77)	(128)	(292)	(12)		(97)	(121)	(250)
6.5	0.4	-	10.2	14.7	34.3	1.8	-	9.1	14.5	31.4	10.1	1.2	-	7.8	16.7	39.9	3.4	-	11.7	17.1	37.3
	(1)		(27)	(39)	(91)	(4)		(20)	(32)	(69)		(9)		(58)	(124)	(296)	(23)		(79)	(115)	(251)
6.9	1.2	-	8.0	14.4	38.8	1.0	-	10.8	12.3	36.0	10.6	1.8	-	7.7	19.7	41.6	2.0	0.1	10.4	17.1	38.8
	(3)		(20)	(36)	(97)	(2)		(22)	(25)	(73)		(14)		(60)	(154)	(326)	(15)	(1)	(77)	(126)	(286)
7.5	0.8	-	7.9	20.3	39.4	0.5	0.5	7.0	20.1	37.2	11.0	2.2	-	8.6	20.6	41.7	2.9	0.7	10.8	19.1	33.8
	(2)		(19)	(49)	(95)	(1)	(1)	(14)	(40)	(74)		(17)		(65)	(156)	(316)	(21)	(5)	(78)	(138)	(244)
7.9	0.8	-	11.6	15.1	42.6	0.5	-	16.2	18.1	37.1	11.6	0.9	0.1	9.1	19.8	47.6	2.8	0.3	18.9	19.2	34.6
	(2)		(30)	(39)	(110)	(1)		(34)	(38)	(78)		(7)	(1)	(68)	(148)	(356)	(20)	(2)	(134)	(136)	(245)
8.5	1.2	-	4.6	12.0	33.6	0.9	-	10.5	18.2	32.3	12.0	1.5	0.1	9.0	17.7	40.9	3.2	0.6	14.2	19.6	30.0
	(3)		(12)	(31)	(87)	(2)		(23)	(40)	(71)		(10)	(1)	(61)	(120)	(277)	(21)	(4)	(93)	(129)	(197)
8.9	1.2	-	14.5	23.8	38.3	2.3	0.9	10.5	14.5	31.8	12.5	1.8	0.1	12.9	22.1	36.5	4.8	1.0	12.1	15.1	28.4
	(3)		(37)	(61)	(98)	(5)	(2)	(23)	(32)	(70)		(13)	(1)	(91)	(156)	(258)	(33)	(7)	(83)	(103)	(194)
9.9	1.2	-	8.6	17.2	41.8	1.9	0.5	8.8	18.1	33.8	13.5	3.6	0.3	12.9	19.2	37.9	10.4	2.3	11.9	14.8	25.0
	(3)		(22)	(44)	(107)	(4)	(1)	(19)	(39)	(73)		(23)	(2)	(82)	(122)	(240)	(63)	(14)	(72)	(90)	(152)
10.9	1.9	-	6.7	20.1	37.2	2.2	0.4	10.4	19.6	28.7	14.5	2.8	-	14.3	18.0	34.4	11.9	2.2	12.0	13.2	23.5
	(5)		(18)	(54)	(100)	(5)	(1)	(24)	(45)	(66)		(20)		(101)	(127)	(243)	(82)	(15)	(83)	(91)	(162)
11.5	1.1	-	7.1	13.5	41.4	3.8	-	10.8	14.8	32.3	14.9	4.9	0.6	11.4	16.0	32.4	15.5	2.7	8.0	12.3	23.7
	(3)		(19)	(36)	(110)	(8)		(24)	(33)	(72)		(32)	(4)	(75)	(105)	(213)	(97)	(17)	(50)	(77)	(148)

*Overweight and obese according to the Cole *et al*. cut-off point (27);

†The international cut-off points for thinness grade 1, 2, and 3 by sex for exact ages defined to pass through BMI of 16, 17, and 18 were used (28,29); MD=Moderate thinness; ML=Mild thinness; OB=Obese; OVW=Overweight; SRV=Severe thinness

From the data presented in Table 2, it was clear that thinness was a much greater public-health problem than overweight. The degree of thinness shifted from severe to mild as the child grew older. Among the preschool boys, severe thinness decreased from 53.7-21.8% at age 4.5-5.5 years to 4.6-14.5% at age over 5.5 years. The same pattern was observed among the girls and primary school children of both sexes. There was a consistent and significant (p<0.05) increase in mild thinness, indicating a regression to the mean as the child grew older.

Table 3 presents the number of subjects at risk at the initial measurements (mean age of 4.5 years for preschool and 8.1 years for primary school children) and tracking coefficient for overweight and thinness (sever, moderate, and mild) risk factors, calculated with GEE over an eight-year period from November 1996 to May 2003. For the preschool children, the odds ratio for overweight and thinness ranged from 1.296 (95% CI 1.219-1.376) to 1.543 (95% CI 1.511-1.578) and was slightly lower compared to the primary school children (odds ratio ranged from 1.385 (95% CI 1.340-1.433) to 1.848 (95% CI 1.749-1.950) for the same variables.

**Table 3.** Number of subjects at risk at initial measurements (mean age 4.5 years for preschool and 8.1 years for primary school children) and tracking coefficient for overweight and thinness (sever, moderate, and mild) risk factors, calculated with generalized estimated equation over an 8-year period from November 1996 to May 2003, Ellisras Longitudinal Growth and Health Study

Malnutrition and gender	Subjects at risk		Odds ratio	p value	95% CI
	No.	%			
Preschool					
Overweight					
Boys	11	3.7	1.543	0.000	1.511-1.578
Girls	12*	4.7	1.618	0.000	1.551-1.685
Severe Thinness					
Boys	158	53.7	1.296	0.000	1.219-1.376
Girls	123	48.4	1.322	0.000	1.232-1.418
Moderate thinness					
Boys	43	14.6	1.267	0.000	1.208-1.328
Girls	34	13.4	1.375	0.000	1.302-1.455
Mild thinness					
Boys	42	14.3	1.296	0.000	1.219-1.376
Girls	32	12.6	1.322	0.000	1.232-1.418
Primary school					
Overweight					
Boys	20	2.2	1.531	0.000	1.487-1.576
Girls	24*	3.1	1.848	0.000	1.749-1.950
Severe thinness					
Boys	393	43.6	1.492	0.000	1.441-1.545
Girls	291	37.5	1.467	0.000	1.418-1.515
Moderate thinness					
Boys	134	14.9	1.421	0.000	1.374-1.470
Girls	95	12.2	1.385	0.000	1.340-1.433
Mild thinness					
Boys	213	23.6	1.492	0.000	1.440-1.545
Girls	199	25.6	1.467	0.000	1.418-1.516

*Obese and overweight children were taken together; CI=Confidence interval

Table 4 shows a specific tracking coefficient from partial correlation controlled for age and maturation between the BMI values at the first measurements and the subsequent measurements. The tracking coefficient was high (ranged from 0.16 to 0.53) and significant (p<0.05 and 0.001) for the first nine measurement sessions and low and insignificant (0.12-0.20) at the last three measurement sessions for both preschool and primary school children. The longitudinal tracking coefficient derived from GEE for BMI of the preschool children ranged from B=-0.01 (95% CI −0.01,-0.001) to B=0.61 (95% CI 0.56-0.66) and of the primary school children B=0.10 (95% CI 0.08-0.12) to B=0.55 (95%CI 0.50-0.59). The longitudinal tracking coefficient estimated from the GEE was low for both preschool and primary school boys and high for girls in both school levels (Table 4). Table 5 shows regression coefficient, 95% CI, and p value in the association of the initial measurements of BMI and the subsequent measurements adjusted for age and maturation. Both preschool and primary school children showed a significant association between the first BMI measurements and the subsequent measurement which ranged from B=0.19 (95% CI 0.05-0.35) to B=0.78 (95% CI 0.64-0.93) for the preschool children B=0.18 (95% CI 0.11-0.26) to B=0.71 (95%CI 0.62-0.79) for the primary school children.

**Table 4.** Specific tracking coefficient (partial correlation coefficient controlled for age and maturation) between the values at the first measurements and the subsequent measurements and tracking or stability coefficient estimated from generalized estimated equation for body mass index for Ellisras rural children

Preschool children			Primary school children		
Mean age (years)	Boys	Girls	Mean age (years)	Boys	Girls
4.9	0.53**	0.46**	8.5	0.48**	0.52**
5.5	0.51**	0.25**	9.1	0.40**	0.39**
5.9	0.42**	0.23**	9.6	0.26**	0.28**
6.5	0.34**	0.18*	10.1	0.24**	0.23**
6.9	0.21**	0.21*	10.6	0.22**	0.20**
7.5	0.23**	0.18*	11.0	0.22**	0.21**
7.9	0.22**	0.19*	11.6	0.20**	0.21**
8.5	0.25**	0.17*	12.0	0.13*	0.22**
8.9	0.21**	0.16*	12.5	0.22**	0.20**
9.9	0.16*	0.15*	13.5	0.13*	0.20**
10.9	0.16*	0.17*	14.5	0.12*	0.18**
11.5	0.20*	0.17*	14.9	0.13*	0.19**
Stability	-0.01	0.61	Stability	0.10	0.55
95% CI	-0.01-0.001	0.56-0.66	95% CI	0.08-0.12	0.50-0.59

*p<0.05;

**<0.001; Stability=Longitudinal tracking coefficient estimated using GEE; 95% CI=Confidence interval; GEE=Generalized estimating equation

**Table 5.** Regression coefficient, 95% confidence interval, and p value controlled for age and maturation in association of initial body mass index measurements with subsequent measurements for Ellisras preschool and primary school children

Mean age (years)	Boys			Girls		
	Beta	p value	95% CI	Beta	p value	95% CI
Preschool children						
4.9	0.68	0.000	0.55-0.82	0.78	0.000	0.64-0.93
5.5	0.67	0.000	0.50-0.84	0.63	0.000	0.43-0.82
5.9	0.58	0.000	0.39-0.77	0.45	0.000	0.24-0.66
6.5	0.50	0.000	0.29-0.70	0.39	0.001	0.16-0.61
6.9	0.26	0.04	0.02-0.49	0.33	0.008	0.09-0.58
7.5	0.37	0.004	0.12-0.62	0.31	0.011	0.07-0.55
7.9	0.39	0.001	0.17-0.62	0.41	0.003	0.14-0.67
8.5	0.26	0.020	0.04-0.49	0.35	0.003	0.12-0.59
8.9	0.22	0.027	0.03-0.41	0.30	0.006	0.09-0.52
9.9	0.28	0.003	0.09-0.46	0.23	0.027	0.03-0.43
10.9	0.22	0.006	0.06-0.37	0.14	0.074	-0.01-0.29
11.5	0.25	0.002	0.09-0.41	0.19	0.010	0.05-0.35
Primary school children						
8.5	0.65	0.000	0.56-0.74	0.71	0.000	0.62-0.79
9.1	0.67	0.000	0.56-0.77	0.66	0.000	0.56-0.75
9.6	0.49	0.000	0.38-0.62	0.48	0.000	0.37-0.58
10.1	0.49	0.000	0.37-0.61	0.43	0.000	0.32-0.53
10.6	0.44	0.000	0.32-0.55	0.39	0.000	0.29-0.49
11.0	0.39	0.000	0.28-0.50	0.35	0.000	0.26-0.45
11.6	0.37	0.000	0.26-0.48	0.36	0.000	0.27-0.44
12.0	0.28	0.000	0.17-0.39	0.28	0.000	0.20-0.37
12.5	0.29	0.000	0.19-0.39	0.28	0.000	0.21-0.35
13.5	0.19	0.000	0.10-0.27	0.21	0.000	0.14-0.28
14.5	0.18	0.000	0.10-0.25	0.19	0.000	0.13-0.25
14.9	0.18	0.000	0.11-0.26	0.17	0.000	0.10-0.23

CI=Confidence interval

## DISCUSSION

To assess the stability of certain variables in time or to assess the predictive value of variables which are measured in early life, the computation of tracking coefficients is considered to be critical in longitudinal epidemiological studies. Recommendations for interpreting tracking correlations are as follows: <0.3=low, 0.3-0.6=moderate, and >0.6=high (37-38). Based on these recommendations, the results of this study suggest that the BMI demonstrated a moderate tracking for both Ellisras rural preschool and primary school children. Tracking coefficient from GEE were high for primary and preschool girls compared to boys.

The adipose rebound and the adolescence period are critical periods for the development of adiposity which poses a threat later in life (12,39). Furthermore, Zafon argues that gains in adipose at key ages are controlled by regulatory mechanism that favour storage of fat when energy is readily available and that high fat mass has important survival values in the face of stressors likely to be encountered during the specific development period (40). The results of the present study have shown the development of BMI for girls to be greater than that of boys from 12 years to 14.9 years which marked individual and group variations among girls due to sexual maturation (11,12,19,39). However, as expected, the mean BMI in the present study was low compared to children in the Bogalusa Heart Study (41), the reference population, and Amsterdam Growth and Health Study children (10) which confirms the developing nature of the African continent compared to other developed countries.

The stability coefficients of BMI between the different age-groups found in our study was statistically significant (p<0.05) and similar to that found by Gasser *et al*. (42) and Fuentes *et al*. (43). The lower stability of BMI observed from the first measurements of both preschool and primary school children to the last three measurements was expected since it covers a long follow-up period of childhood and late adolescence. A similar low correlation level of BMI emanating from the long follow-up period between childhood and adolescence or young adulthood has been reported (42,44-48), indicating that the accumulations of fat in the pre-pubertal period may prepare an individual for the high energy needs of the pubertal growth spurt and reproduction.

Linear regression analysis confirms the tracking of BMI during childhood and adolescence found in the correlation analysis. First, age and maturity were a good predictor of BMI at an early age for both preschool and primary school children as reported earlier in other studies (12,39,49-50). This may reflect the association between maturity, age, and the adiposity during childhood and adiposity rebound during adolescence with girls having high BMI from 7.9 years (13.8 kg/m^2^) for the preschool and from 11.6 years (14.8 kg/m^2^) for the primary school children. Interestingly, girls continued high adiposity up to 11.5 years (15.4 kg/m^2^) for the preschool and to 14.9 years (18 kg/m2) for the primary school children. This is different from the findings of the study by Fuentes *et al*. (42), Malina (37), and Marshall *et al*. (38) who showed that girls having lower BMI during infancy and boys having a later adiposity rebound during childhood. Boys and girls showed a similar BMI level at 15 years in the sample of Fuentes *et al*. (43) while in the current sample girls exhibited a significant high BMI than boys.

The prevalence of overweight for both preschool children (0-3.9%) and primary school children (0-15.5%) was low compared to 11-15% among 6-11 years old children and from 11% to 16% among 12-19 years old children of the National Health and Nutritional Examination Survey IV (50). Furthermore, the prevalence of thinness was high in the present sample even during the adipose rebound (39.4-42.6%) and the adolescence stage (23.7-30.0%) which clearly shows lean mass than fat mass (28). The increasing prevalence of thinness which is an indication of undernutrition supported by significant odds ratio (1.267 (95% CI 1.208-1.376) to 1.8 (95% CI 1.749-1.950) raises a serious concern as relative lean young black females appears to mature into relative obese black women (49).

Although no empirical data are available on nutritional intake and food practices in these villages, one (KDM) of the current authors lived in this area throughout childhood and remembered that girls spend their time in and around the house and would be likely to obtain food they, their mothers, grandmothers, or other female siblings prepare. During sporadic occasions (month end, mirages, funerals, etc.), high-energy food is available in most Elliras rural families. The capability of boys to become independent goat and cattle herders coincides with their school attendance. They return from school each afternoon to carry out their herding chores. During this time, their nutritional intake is likely to deteriorate because they are distanced from the preparation and source of food, which is unlikely to be kept for them on their return. The boys, usually aged about 7-15 years, rely on gathering vegetable foods (berries, etc.), trapping rabbits and hares, and/or killing birds which they cook and eat during the day. Therefore, they do not have a guaranteed daily nutritional intake. The staple family diet is porridge (mealie-meal/maize) which is prepared daily in most families by female siblings or parents. This porridge remains in the cooking-pot for members of the family to serve themselves when they are hungry. In common with data on Pedi children reported by Steyn *et al*. (51), it may be that these children, who are mainly from Tswana origin, have two meals a day with a very low energy intake. While the above description is anecdotal and may be viewed as having a little scientific basis, such reports of dietary behaviour regarding children in Southern Africa are rare in the literature and, therefore, should not be ignored as a possible explanation of rural South African children's nutritional intake. Furthermore, the physical activity and physical fitness level of the present sample was not part of our analysis. Monyeki *et al*. reported low physical activity patterns among girls compared to boys (52).

The assessment of breast development was also a problematic. Although we were able to obtain a visual assessment of breast development rather than relying on self-reports from girls, fat tissue can be mistaken for breast tissue in cases where the breast is not palpated. However, a key advantage of this method is that it is widely used by researchers and clinicians, thereby increasing its applicability. Physical activity and physical fitness of these children were not controlled for in the analysis. Furthermore, the use of BMI as an indicator of fatness in childhood has been questioned because of its dependency on gynaecological age (in contrast to chronological age), since the body composition and body frame change rapidly during growth and puberty (53). However, recently, the International Obesity Task Force concluded that BMI is a reasonable measure with which to assess overweight in children and adolescents (54). The dependency of BMI on age is likely not to be a source of bias in this study because a cohort of children aged 3-10 years was examined at the same time during the follow-up period.

In conclusion, we found a significant tracking of BMI as children grew older which was more consistently shown for the preschool girls (4.5-11.5 years) and for the primary school girls (8.1-14.9 years). The development of obesity and overweight was more prevalent among both preschool and primary school girls compared to boys while for thinness it was high for both preschool and primary school children. Investigation of nutritional intake and physical activity patterns will shed light on how healthy these children are and their lifestyles. Community awareness on healthy lifestyle may have a key role in the prevention of obesity late in life.

## ACKNOWLEDGEMENTS

The financial support received for the ELS from the VU University Medical Center, the University of Limpopo, South Africa, the National Research Foundation, and the Medical Research Council of South Africa is appreciated. The authors are indebted to the ELS administrators Mr. T.T. Makata, L. Majadibodu, and U.T. Motlogeloa for coding the ELS data. Monyeki MS and Malatji MJ (Makgoka High School, Limpopo province) are thankfully acknowledged for editing this manuscript.
